# Extensive Clinical Flow Cytometric Lymphocyte Phenotyping in Myasthenia Gravis: A Single‐Center Study

**DOI:** 10.1111/jnc.70126

**Published:** 2025-06-17

**Authors:** Hannes Lindahl, Malin Petersson, Sara Lind Enoksson, Fredrik Piehl, Susanna Brauner

**Affiliations:** ^1^ Department of Clinical Immunology and Transfusion Medicine Karolinska University Hospital Stockholm Sweden; ^2^ Department of Clinical Neuroscience Karolinska Institutet Stockholm Sweden; ^3^ Department of Clinical Science, Intervention and Technology Karolinska Institutet Stockholm Sweden; ^4^ Department of Neurology Karolinska University Hospital Stockholm Sweden; ^5^ Neuroimmunology Unit Center for Molecular Medicine, Karolinska Institutet Stockholm Sweden

**Keywords:** CD4 T cells, flow cytometry, myasthenia gravis, prediction model, prognosis

## Abstract

Myasthenia gravis (MG) is an autoimmune neurological disease characterized by potentially life‐threatening muscular fatiguability. Symptoms are directly linked to autoantibodies targeting postsynaptic receptors of the neuromuscular junction. However, the underlying immunopathogenesis remains to be elucidated. This single‐center study aimed to characterize peripheral blood lymphocytes in MG patients and to identify prognostic biomarkers. We retrospectively reanalyzed clinical flow cytometric data on blood B and T cells from 76 incident MG cases, comparing them to healthy individuals of relevant age range. Clinical data was collected from the Swedish MG registry and used for outcome analyses. Flow cytometry analyses were based on standardized panels established by the Human Immunology Project Consortium and included a 10‐color T cell panel focused on memory, polarization, and activation states and an 8‐color B cell panel that includes memory phenotypes, transitional B cells and plasmablasts. Groupwise comparisons, survival curves, and regression models adjusting for potential confounders were used to assess potential predictors of the primary outcome, minimal disease manifestation within one year. Untreated MG patients had higher frequencies of cluster of differentiation 4 (CD4) T cell frequencies compared to healthy individuals of approximately the same age (median 75% vs. 63% in age range 40–59 years and 65% in 60–81 years). High CD4 T cell frequencies (> 75% of total T cells) were associated with lower probability of minimal disease manifestation within one year (35% vs. 67%; log rank *p* = 0.032). In a multivariable Cox regression model assessing time to minimal disease manifestation, CD4 T cell frequency was an independent risk factor (*p* = 0.0014). In conclusion, MG patients appear to display an altered CD4 T cell phenotype and frequency of CD4 T cells is a potential prognostic biomarker.

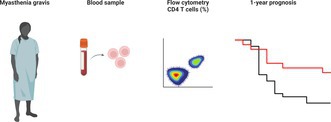

## Graphical abstract

Myasthenia gravis, an autoimmune neurological disorder causing severe muscular fatiguability, involves autoantibodies targeting neuromuscular junction receptors. This study analyzed peripheral blood lymphocytes from 76 MG patients to identify prognostic biomarkers. Results highlight altered CD4 T cell phenotypes and suggests CD4 T cell frequency as a potential prognostic biomarker of disease severity.

**Abbreviations**
AChR: acetylcholine receptor
CD: cluster of differentiation
CI: confidence interval
EDTA: Ethylenediaminetetraacetic acid
EOMG: early onset MG
HLA: human leukocyte antigen
HR: hazard ratio
IVIG: intravenous immunoglobulin
LOMG: late onset MG
LRP4: low‐density lipoprotein receptor‐related protein 4
MG: myasthenia gravis
MuSK: muscle‐specific tyrosine kinase
NK: natural killer
QMG: quantitative MG
ROC: receiver operating characteristics
RRID: research resource identifier (see scicrunch.org)
TAMG: thymoma associated MG

## 1 Introduction

Myasthenia gravis (MG) is an antibody‐mediated autoimmune disease characterized by fatigable muscle weakness. Most MG patients have autoantibodies directed against the nicotinic acetylcholine receptor (AChR) in the postsynaptic membrane of the neuromuscular junction, with smaller proportions being positive for autoantibodies against muscle‐specific kinase (MuSK), low‐density lipoprotein receptor‐related protein 4 (LRP4) or are seronegative to all three antigens (Gilhus et al. 2019). Although treatments targeting humoral immunity, such as plasma exchange, inhibitors of neonatal Fc receptors, and B cell depletion, are effective in MG (Piehl et al. 2022), antibody titers per se do not correlate well with disease severity on the MG population level (Piehl et al. 2022; Brauner et al. 2020; Roses et al. 1981; Sanders et al. 2014). In fact, T cell subsets have recently been linked to disease severity and prognosis (Ingelfinger et al. 2021; Ashida et al. 2021), arguing for a more complex immunological background.

The thymus is a primary lymphoid organ essential for eliminating self‐reactive T cells, and it hence plays a vital role in gatekeeping the immune system. Previous studies have suggested pathogenic mechanisms in both central and peripheral tolerance in MG (Liu et al. 2014; Xu et al. 2020; Thiruppathi et al. 2012; Michelson et al. 2022; Renton et al. 2015) and thymic pathology is closely linked to MG disease development (Wolfe et al. 2016). In approximately 10%–15% of cases, MG is associated with a tumor of the thymus (thymoma‐associated MG, TAMG), while most early‐onset MG patients (EOMG; disease onset < 50 years) display thymic hyperplasia characterized by germinal centers, in which T and B cells are believed to interact to propagate disease‐driving immune responses (Berrih‐Aknin et al. 1987). However, the role of T cells in MG pathogenesis is far from clear, and no defined T cell‐marker correlating with clinical outcomes has been established in clinical routine.

Most MG patients require chronic immune modulatory treatment to achieve acceptable symptom status. The benefit–risk balance with different therapeutic options varies, and an escalation approach has so far been common practice (Narayanaswami et al. 2021). However, recent evidence suggests that treatment responses are superior with early treatment compared with later in the disease (Piehl et al. 2022; Uzawa et al. 2023a, 2023b). In addition, early intervention with anti‐cluster of differentiation (CD)20 treatment has been shown to reduce the risk of later hospitalization for MG worsening (Piehl et al. 2022). In this context, biomarkers predicting disease severity could serve to help establish individualized treatment strategies. In addition, if such biomarkers rely on established technologies, such as flow cytometry, clinical implementation is greatly facilitated. In this study, we retrospectively analyzed all clinical routine flow cytometry T and B cell panels performed at the Karolinska MG center from January 2017 to April 2023 together with longitudinal clinical data. The objective was to explore MG disease‐associated changes in the T and B cell compartments compared to a healthy reference population and to identify potential prognostic biomarkers.

## 2 Methods

### 2.1 Patients and Data

Patients with MG were identified through the Swedish MG‐registry, a nationwide patient quality registry only containing patients with a physician‐confirmed diagnosis. The coverage of the MG registry is > 80% in the Region of Stockholm, thus representing a population‐based sample (Piehl et al. 2022). Clinical baseline and outcome data, including disease subtype, serology, clinical manifestations, disease activity scores, and treatment regimen were collected from the registry. Disease severity was assessed using the modified quantitative MG (QMG) score (Besinger et al. 1983). Minimal disease manifestation was defined as QMG score ≤ 4p and prednisolone dose ≤ 10 mg daily, without rescue treatment within the previous 3 months. Rescue treatment was defined as pulse of prednisone, methyl prednisone, prednisolone doses > 40 mg daily, or pulse intravenous immunoglobulin (IVIG) or plasmapheresis. Active disease at baseline was defined as a QMG score ≥ 5p. Wash‐out periods after discontinuation of immunomodulatory drugs were set to 3 months.

All flow cytometry samples analyzed during the period January 2017 to April 2023 at the Department of Clinical Immunology and Transfusion Medicine at Karolinska University Hospital were cross‐referenced with patients included in the MG‐registry. In total, 135 samples were identified, of which 28 were excluded as sampling was undertaken for reasons other than diagnostic workup or monitoring of MG, for example, ongoing infection or hematologic disease. Two samples from MuSK‐positive patients were excluded as the immunopathogenesis in this MG subtype is generally regarded as distinctly different. The remaining 105 samples were from 76 unique patients with a definitive diagnosis of MG, which had been investigated using either a 10‐color (*n* = 80) or a 5‐color (*n* = 6) flow cytometry peripheral blood T cell phenotyping panel, or an 8‐color B cell panel (*n* = 59). Concentrations of T, B, and natural killer (NK) cells were assessed in all samples.

### 2.2 Sample Collection and Flow Cytometry

A volume of 2–5 mL of blood was sampled by venipuncture into standard EDTA blood collection tubes and were stored at room temperature < 24 h prior to sample pre‐processing and flow cytometry analysis. Total T, B, and NK cell concentrations were determined by a commercial automated system using the Tetra‐2 panel (B23534, Beckman Coulter; Brea, CA, US) on an Aquios CL flow cytometer (Beckman Coulter). Whole blood was loaded into the machine, where pipetting, erythrocyte lysis, staining, and gating is automated and set by the manufacturer. Antibodies used in this system, that is, the Tetra‐1 (CD4 and CD8 T cells, B23533, Beckman Coulter) and Tetra‐2 (T, B, and NK) panels, were (all mouse anti‐human and from Beckman Coulter): CD45‐FITC (clone: B3821F4A), CD4‐RD1 (clone: SFCI12T4D11), CD8‐ECD (clone: SFCI21Thy2D3), CD3‐PC5 (clone: UCHT1), CD56‐RDI (clone: N901), and CD19‐ECD (clone: 3G8/J3‐119). CD4 and CD8 T cell concentrations were derived from T cell subset frequencies generated in the flow cytometric panels and total T cell concentrations. In six cases absolute CD4 and CD8 concentrations were determined with the Tetra‐1 panel (Beckman Coulter).

Specific T and B cell phenotyping was performed based on standardized panels established by the Human Immunology Project Consortium (Maecker et al. 2012). Samples were handled manually, and whole blood was subjected to red cell lysis using IO‐test 3 Lysing solution (A07799, Beckman Coulter) according to the manufacturer's instructions. A 10‐color panel was used to quantify percentages of T cell subsets. Custom‐ordered Lyotubes (Becton Dickinson, Franklin Lakes, NJ, US) were used with a lyophilized antibody cocktail consisting of: CCR6‐BB515 (clone: 11A9, RRID: AB_2738825), CCR7‐PE (clone: 150503, RRID:AB_2033949), CXCR3‐PE‐CF594 (clone: 1C6/CXCR3, RRID: AB_11153118), CD4‐PerCP‐Cy5.5 (clone: RPA‐T4, RRID:AB_1727476), CD45RA‐PE‐Cy7 (clone: HI100, RRID:AB_1727498), CD38‐APC (clone: HIT2, RRID:AB_398599), CD45‐Alexa Fluor 700 (clone: HI30, RRID:AB_1645452), CD8‐APC‐H7 (clone: SK1, RRID:AB_1645481), CD3‐V450 (clone: UCHT1, RRID: AB_1645570), and HLA‐DR‐V500 (clone: L243, RRID:AB_10563765). Additionally, the 8‐color B cell panel used for B cell subsets included the following antibodies: IgD‐FITC (clone: IA6‐2, RRID:AB_396113), CD21‐PE (clone: BL13, A32536, Beckman Coulter), CD19‐ECD (clone: J3‐119, A07770, Beckman Coulter), CD27‐PC7 (clone: 1A4CD27, RRID:AB_2934286), CD20‐APC‐A700 (clone: B9E9, B12112, Beckman Coulter), CD38‐APC‐A750 (clone: LS198‐4‐3, B49200, Beckman Coulter), IgM‐Pacific Blue (clone: SA‐DA4, B30656, Beckman Coulter), and CD45‐Krome Orange (clone: J33, RRID:AB_2888654). Results were compared to a previously published healthy reference population (Oras et al. 2020). The gating strategy is included in the Supporting Information material as Figure S1.

### 2.3 Data Analysis and Statistics

As this was an exploratory study using secondary data, no formal sample size calculation was performed. However, based on prior experience from similar studies, the clinical center's coverage area was considered sufficient for the planned analyses. For the analysis of MG patients not exposed to immune modulation, repeated measurements from the same patient were averaged. When assessing the effect of immune modulation on lymphocyte subsets, all blood samples were treated as independent observations. However, if a patient had multiple samples within the same treatment category, only the most recent sample was retained. A minimum of five data points were required for a treatment category to be included in the analysis. For prediction of prognosis, each patient was represented only with their earliest sample, with a minimum clinical follow‐up of 12 months. Intra‐individual variability was summarized as the median fold change from the first sample. This analysis was limited to the large T cell panel, including B and NK cell quantification, as it had the highest number of patients with repeated measurements (*n* = 15). Lymphocyte subsets were not assumed to follow a normal distribution, and non‐parametric descriptive statistics and hypothesis testing were used. Receiver operating characteristic (ROC) curve analysis was performed to identify potential predictive biomarkers. Kaplan–Meier analysis was used to assess time to minimal disease manifestation in relation to biomarker status, with statistical significance determined by the log‐rank test. Patients who did not achieve minimal disease manifestation within one year were right censored at this time point. To evaluate the independent contribution of selected predictors on time to minimal disease manifestation, multivariable Cox regression was performed. Throughout, *p*‐values were adjusted for multiple comparisons using the Bonferroni method and *p* < 0.05 was considered statistically significant. No test for outliers was conducted. All analyses and figures were generated using R v 4.3.1 (the Comprehensive R Archive Network project) with RStudio 2023.06.1 + 524.

## 3 Results

### 3.1 Study Population and Clinical Characteristics

We included results from 105 flow cytometry analyses from 76 unique patients (Table 1). The majority of patients were sampled once and 21 patients were sampled more than once. Characteristics associated with samples and patients are detailed separately. The median age at onset of MG was 57 years (IQR 38–68), 62% met the criteria for Late‐Onset MG (LOMG), defined as onset at age ≥ 50 years without thymoma, and 45% were female. A significant majority (91%) had tested positive for AChR antibodies, while the remaining were seronegative. Among all samples, the median disease duration at the time of sampling was 1.6 years and 43 samples (41%) were collected without ongoing immune modulation, as part of initial diagnostic workup.

**TABLE 1:** Sample and patient characteristics.

	All blood samples (*n* = 105)	All patients (*n* = 76)	Untreated patients (*n* = 42)
Female, *n* (%)	46 (44)	34 (45)	20 (48)
Age at onset, median (IQR)	57 (40–67)	57 (38–68)	61 (49–69)
Age at sampling, median (IQR)	62 (49–73)	65 (50–74)	63 (51–76)
Subtype, *n* (%)			
EOMG	28 (27)	22 (29)	9 (21)
LOMG	64 (61)	47 (62)	32 (76)
TAMG	13 (12)	7 (9)	1 (2)
oMG, *n* (%)	9 (9)	7 (9)	3 (7)
Serology, *n* (%)			
AChR‐Ab	98 (93)	69 (91)	39 (93)
Seronegative	7 (7)	7 (9)	3 (7)
Thymectomized, *n* (%)	37 (35)	25 (33)	10 (2)
Disease duration at sampling, years, median (IQR)	1.6 (0.5–8.7)	2.8 (0.3–9.7)	1.5 (0.4–7.6)
QMG score at sampling, median (IQR)	6 (2–9)	6 (3–9)	7 (2–10.5)
Treatment, *n* (%)			
No immune modulatory treatment	43 (41)		
Corticosteroids	36 (34)		
IVIG	19 (18)		
Rituximab	16 (15)		
Azathioprine	10 (10)		
Ciclosporin	2 (2)		
Mycophenolate mofetil	2 (2)		
Tocilizumab	2 (2)		
Cyclophosphamide	1 (1)		

*Note:* Characteristics of included samples and patients are summarized separately because 21 patients were represented with two or more samples. To obtain summary statistics for disease duration and severity for the included patients, earliest values of repeated sample timepoint were used. Numbers of blood samples taken during, or within 3 months of stopping, MG treatment with specified Immune modulatory agents are listed. Generalized MG (gMG) is assumed if not classified as ocular MG (oMG).

Abbreviations: AChR‐Ab, acetylcholine receptor antibody; EOMG, early onset MG; IQR, interquartile range; IVIG, intravenous immunoglobulin; LOMG, late onset MG; *n*, number of samples or participants; QMG, quantitative MG; TAMG, thymoma associated MG.

### 3.2 Circulating Lymphocyte Subsets in Untreated Patients

**FIGURE 1:**
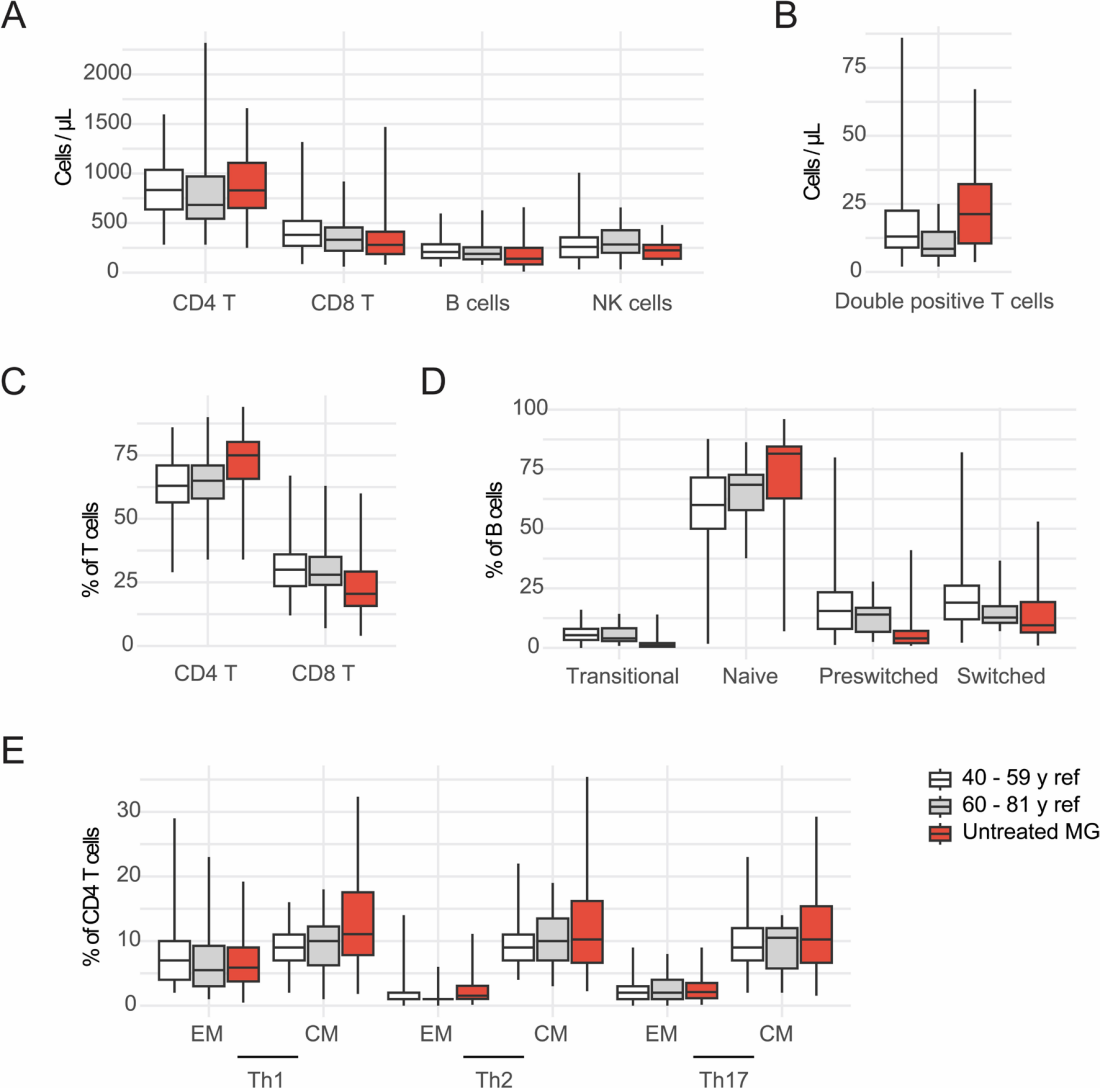
Two healthy reference populations compared with untreated MG patients. Summary statistics from previously reported healthy populations with relevant age ranges (40–59 years, *n* ≥ 99 and 60–81 years, *n* ≥ 18) were compared with MG patients sampled without any immunomodulatory treatment (*n* = 42). Boxplots show the median (line), interquartile range (box), and data spread (whiskers). Lymphocyte subset concentrations (A‐B) and frequencies (C‐E) are shown. T helper (Th)1 is defined as CD4 T cells that are CXCR3 +^+^ CCR6 –^−^, Th2 as CXCR3–^−^ CCR6 +^−^, and Th17 as CXCR3 +^−^ CCR6 +^+^. CM, central memory T cells; EM, effector memory T cells; *n*, number of participants.

We first aimed to identify lymphocyte abnormalities specifically associated with MG. To do so, we compared untreated MG patients (*n* = 42) with a published healthy reference population stratified into two relevant age groups: 40–59 and 60–81 years (Table 2) (Oras et al. 2020). Using summary statistics from Oras et al. (2020), we assessed medians and IQRs across all lymphocyte subsets (Table 2, Figure 1). Untreated MG patients exhibited relatively low CD8 T cell concentrations compared to both reference groups (280 vs. 381 and 331.5 cells/μL; untreated MG vs. controls 40–59 years and 60–81 years). Interestingly, CD4^+^CD8^+^ T cell concentrations appeared elevated (21,3 vs. 13 and 8.5 cells/μL), whereas B cell concentrations appeared reduced (140 vs. 208 and 189.5 cells/μL). Regarding T cell subset proportions, we observed a notable increase in CD4 T cell percentages compared to both reference groups (75% vs. 63% and 65%). Among memory T cell polarization states, central memory Th1 (CXCR3^+^ CCR6^−^) were most prominently associated with untreated MG patients (11.9% vs. 9% and 10%). Aberrations in the B cell compartment included an increased proportion of naïve B cells (81.5% vs. 60% and 68.5%) and a lower proportion of preswitched memory B cells (4% vs. 15.5% and 14%) and switched memory B cells (8.5% vs. 19% and 12.8%).

**TABLE 2:** Lymphocyte subsets in MG patient strata and two healthy reference populations.

	MG, untreated		Healthy, age 40–59 years		Healthy, age 60–81 years		MG, minimal disease manifestation within one year			
	(*n* = 42)		(*n* ≥ 99)		(*n* ≥ 18)		Yes (*n* = 27)		No (*n* = 25)	
	Median	IQR	Median	IQR	Median	IQR	Median	IQR	Median	IQR
T cells/μL	1205	903–1530	1322	1046–1685	1075	884–1492	1130	590–1660	1150	765–1425
CD4 T cells/μL	830	653–1108	834	638–1037	683.5	544–970	690	390–1000	780	615–1135
CD8 T cells/μL	280	188–413	381	270–522	331.5	221–456	220	160–570	220	165–345
DP T cells/μL	21.3	10.5–32.3	13	9–22.5	8.5	6–14.8	17.4	7.8–29.9	19.8	10–39.6
B cells/μL	140	83–250	208	147–286	189.5	134–255	135	68–320	170	75–255
NK cells/μL	225	140–280	259	156–355	284	200–427	175	70–253	190	120–260
CD4 T, % of T cells	75	65.8–80.3	63	56.5–71	65	58–71	69	57–76	76	69–84
Naïve, % of CD4 T	43	24.5–49.5	39	30–49	43	30–48	35	23.5–51.5	40.5	24–49.8
EM	14	11.5–18	16	11—23	19	11—23	15	12.5–19	13	10.5–17.5
CM	41	34–54	40	31–46	44	30–51	48	32.5–58	42	37–52.5
EMRA	1	0.5–2.5	1	0–3.5	1	0–3	1	0.5–3.5	1	0.5–2
Activated	2	1—3	1	1—2	2	1—3	2	1—3	2	1—3
Th1 EM, % of CD4 T	5.8	3.9–7.8	7	4—10	5.5	3–9.3	5.8	4.9–8.6	5	3.3–6.9
Th1 CM	11.9	8.7–14.6	9	7—11	10	6.3–12.3	10.9	8.5–14.4	12.3	7.9–15.2
Th2 EM	1.6	1.16–2.4	1	1—2	1	1—1	1.3	0.7–3.3	1.3	1–2.8
Th2 CM	10.8	7.3–14.7	9	7—11	10	7–13.5	10.8	8.8–15.5	11.5	7.8–14.9
Th17 EM	2.1	1.4–2.8	2	1—3	2	1—4	1.8	1.4–2.5	1.9	1.1–3
Th1 CM	11	7.7–13	9	7—12	10.5	5.8–12	10.1	7.1–15.1	11.7	7.7–15.4
CD8 T, % of T cells	20.5	15.8–29.3	30	23.5–36	28	24–35	25	20–37.7	19	12–26.5
Naïve, % of CD8 T	23	13–37.5	29	19–41	19	11–31	20	8–43	26.5	18.5–35.5
EM	23	18–27.5	19	14–28	22	13–31	21	12–41.5	24.5	19.5–28
CM	12	8.5–19	10	6—15	10	6—12	11	6—16	13	10.3–25.5
EMRA	30	18–46.5	39	20–49	40	29–60	37	18–58	28	14.3–45.3
Activated	5	1.5–10	3	1—5	4	2—7	6	3–9.5	5	1.3–8
DP, % of T cells	1.9	1.1–3.1	1	1—2	1	0–1	1.4	0.9–2.5	2.1	1.2–3.5
Transitional, % of B	1	0.5–2	5.35	3.3–8	4	2.9–8.3	0.5	0.5–1	1	0.5–2
Naïve	81.5	62.8–84.5	60	50–71.5	68.5	57.8–72.7	51	48–84	65	44–87
Preswitched memory	4	2–7.1	15.5	8–23.3	14	6.7–16.8	5	3—20	5	3—20
Switched memory	9.5	6.5–19.3	19	12–26.1	12.8	10.5–17.5	20	7—28	18	5—24
Plasmablasts	0.5	0.5–1	0.53	0.2–1	0.4	0.2–0.8	0.5	0.5–1	0.5	0.5–1

*Note:* Peripheral blood flow cytometry results from untreated myasthenia gravis (MG) patients (*n* = 42 for T, B, and NK cell concentration = 35 for CD4 and CD8 T cells and their memory and polarization states; and *n* = 24 for B cell subsets) and two previously published healthy reference populations. All MG patients with active disease at sampling (*n* = 52) are included stratified by the main clinical outcome, minimal disease manifestation within one year from sampling. T helper (Th)1 is defined as CD4 T cells that are CXCR3^+^ CCR6^−^, Th2 as CXCR3^−^ CCR6^−^, and Th17 as CXCR3^−^ CCR6^+^. Activated T cells are defined as HLA‐DR^+^ CD38^+^. Preswitched memory B cells are defined as CD19^+^ cells that are IgD^+^ CD27^+^, plasmablasts as CD19^+^ cells that are IgM^−/+^CD38^++^, transitional B cells as CD19^+^ cells that are IgM^++^ CD38^++^.

Abbreviations: CM, central memory T cells; EM, effector memory T cells; EMRA, CD45RA^+^ EM T cells; IQR, interquartile range; *n*, number of participants.

### 3.3 Immune Modulation and Circulating Lymphocyte Subsets

To explore the effects of specific MG treatments on lymphocyte subsets, we analyzed samples taken from patients treated with a single immune modulatory treatment (Figure S2). Interestingly, most MG treatments reduced absolute numbers of CD4 T cells. Azathioprine, a first‐line immunosuppressant drug, had a broad suppressive effect on T, B, and NK cell numbers. No effect of these drugs on T cell memory subset or T cell polarization percentages was observed. Activated T cells, defined as HLA‐DR^+^ CD38^+^, were generally increased in MG patients on immune modulatory treatment, especially with IVIG. B cell depleting treatment with rituximab not only reduced B cell numbers but seemed to induce a phenotypic shift characterized by proportionally less switched memory B cells and more transitional and naïve B cells, similar to what has been observed in multiple sclerosis (Figure S2B,H) (Starvaggi Cucuzza et al. 2023). Average time from last rituximab dose and sampling was 10 months, which may explain the relatively high B cell counts.

### 3.4 Stability of Individual T Cell Results Over Time

**FIGURE 2:**
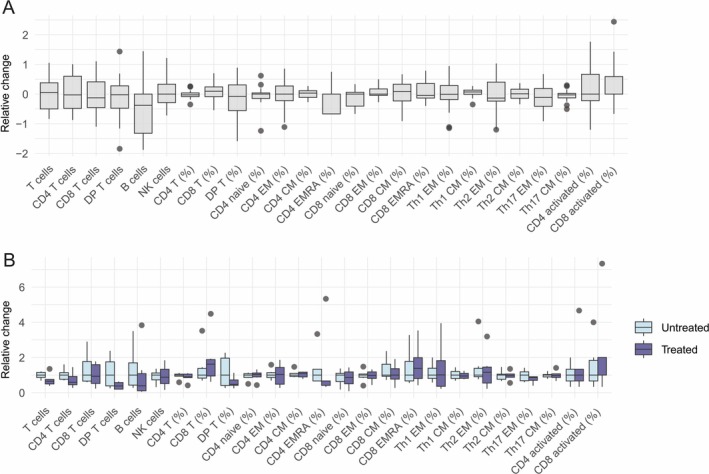
Intra‐individual variability of T cell subsets. Fifteen patients were sampled on two separate occasions (median 176 days apart) for T cell phenotyping including B and NK cell quantification. The intra‐individual variation for each cell population is shown (A). A subset (*n* = 6) of these 15 patients were untreated at the first sample timepoint and were on immunomodulatory MG treatment [IVIG (*n* = 1), IVIG and prednisolone (*n* = 3), and rituximab and prednisolone (*n* = 2)] at the second timepoint. The differences observed when comparing these paired, untreated and treated, samples are shown (B). Boxplots show the median (line), interquartile range (box), and data spread (whiskers); outliers appear as points. T helper (Th)1 is defined as CD4 T cells that are CXCR3^+^ CCR6^−^, Th2 as CXCR3 +^−^ CCR6^−^, and Th17 as CXCR3^−^ CCR6^+^. Activated T cells are defined as HLA‐DR+^+^ CD38 +^+^. DP T, CD4 +^+^ CD8 +^+^ double positive T cells; CM, central memory T cells; EM, effector memory T cells; EMRA, CD45RA^+^ EM T cells; n, number of participants.

Deep T cell phenotyping was performed on more than one occasion for 15 patients, allowing for an assessment of intra‐individual variability in the T cell compartment over time. Fold changes between the first and second samples (collected a median of 176 days apart) are presented as boxplots (Figure 2A). While absolute T cell counts varied, the percentage of CD4 T cells remained remarkably stable within individuals (coefficient of variation = 6.9%). Intra‐individual variability reflects a combination of treatment effects, disease dynamics, and other influencing factors. To specifically examine the impact of immunomodulatory treatment, we analyzed a subset of six patients who had one sample taken before initiating MG treatment and a second sample once treatment had begun (Figure 2B). Treatments included IVIG (*n* = 1), IVIG + prednisolone (*n* = 3), and rituximab + prednisolone (*n* = 2). While absolute counts of lymphocytes were lower following treatment, relative changes in T cell subsets did not exhibit a consistent pattern.

### 3.5 CD4 T Cells and Prognosis

**FIGURE 3:**
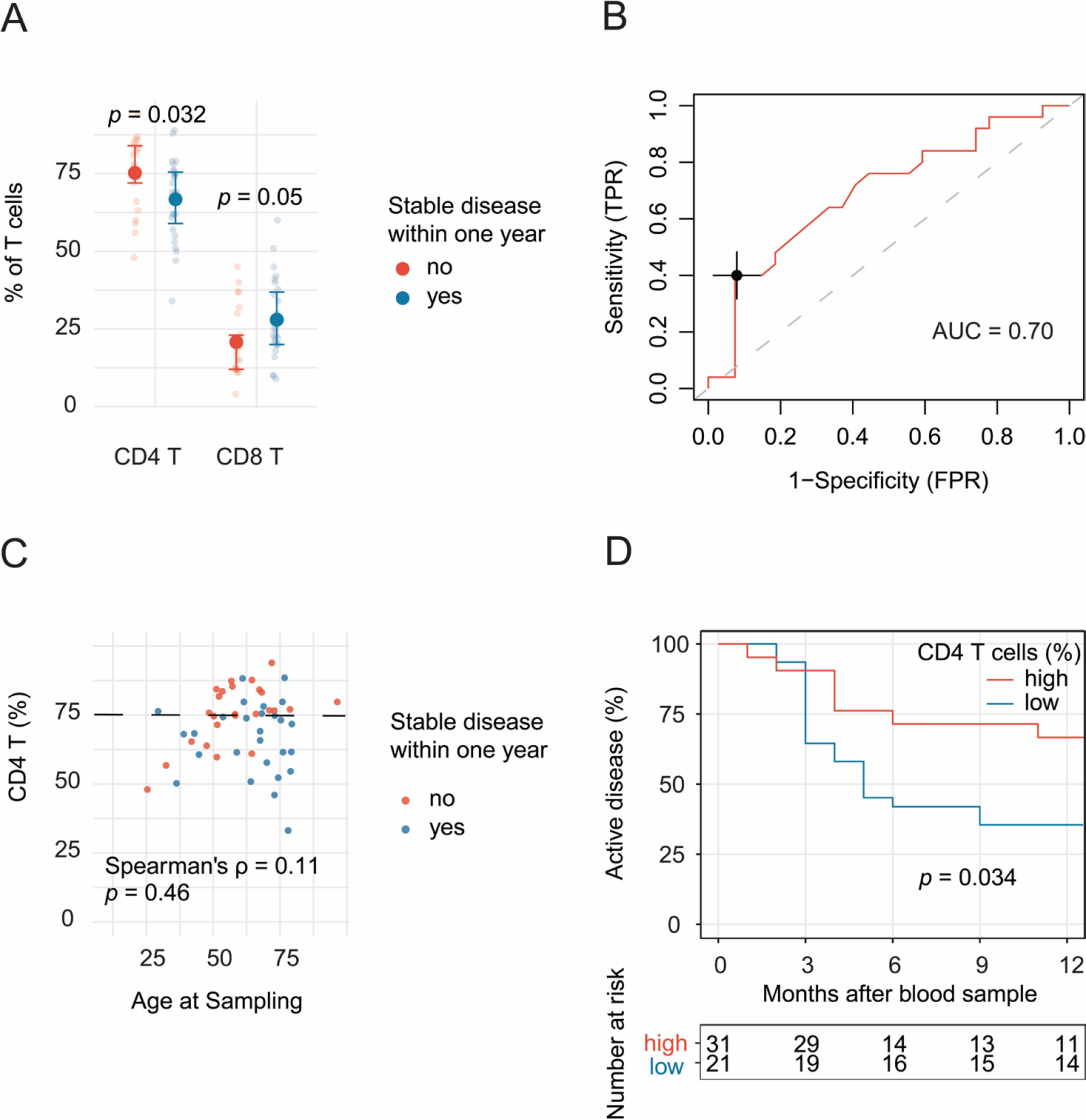
CD4 T cell percentage is associated with time to minimal disease manifestation in MG. Peripheral blood CD4 T cells as a percentage of total T cells were quantified in unique MG patients with active disease, if several samples were available only the earliest was considered. (A) Results for patients that had active disease (*n* = 27, red) were compared with those who had minimal disease manifestation (stable disease, *n* = 25, blue) one year after sampling. Student's *t*‐test was used. Mean and standard error of the mean is indicated. (B) Receiver‐operating characteristics (ROC)‐curve analysis was performed using the results from (A) to assess the discriminative performance of CD4 T cell percentage to predict which patients will exhibit minimal disease manifestation (stable disease) within one year from sampling. Youden's index is indicated corresponding to a CD4 T cell percentage of 75. (C) CD4 T cell percentage is plotted against age at sampling including Spearman's correlation analysis to explore this potential confounder. (D) Kaplan–Meier curve analysis was performed comparing time to minimal disease manifestation with *χ* ^2^ = 4.5 and degrees of freedom = 1. The patients were dichotomized based on having high (> 75%) or low (≤ 75%) CD4 T cell percentage and the curves were compared using the log rank test. *n*, number of participants.

Next, we explored lymphocyte subsets as potential predictors of disease course, focusing on the primary outcome: minimal disease manifestation within one year of sampling. For this analysis, we included samples taken during active disease (*n* = 52), selecting the earliest sample when multiple were available for the same patient. At the time of sampling, immune modulatory treatments were as follows: none (*n* = 23), glucocorticoids (*n* = 10), azathioprine (*n* = 5), IVIG (*n* = 5), mycophenolate mofetil (*n* = 1) and various combinations (*n* = 8). Among patients who achieved minimal disease manifestation within one year, 24 out of 27 were started on a new immune modulatory treatment during follow‐up, most commonly rituximab (*n* = 22). Among those who did not achieve minimal disease manifestation within one year, 15 out of 25 started a new treatment; also here, rituximab was the most commonly used (*n* = 13). The most promising biomarker for achieving minimal disease manifestation within one year was the frequency of total CD4 T cells (Figure 3A; Table 2).

A ROC curve analysis was performed to assess the discriminative ability of CD4 T cell frequency, yielding an area under the curve (AUC) of 0.70, indicating moderate accuracy (Figure 3B). Youden's index identified an optimal cutoff at 75% CD4 T cells, corresponding to a sensitivity of 67% and a specificity of 64%. Next, we stratified the patients at this cutoff and compared time to remission between the two groups (CD4 T cell high vs. low) using Kaplan–Meier survival analysis (Figure 3D). Patients with CD4 T cell frequencies of 75% or lower at the time of sampling were more likely to achieve minimal disease manifestations within one year compared to those with higher frequencies (*p* = 0.034). At 12 months, 67% (95% CI: 49–90) of patients in the CD4 T cell high group still had active disease, whereas only 33% (95% CI: 22–57) remained with active disease in the CD4 T cell low group. Clinical characteristics of this patient subset are presented in Table S2. Further, as some studies have shown that CD4 T cell percentage increases with age (Oras et al. 2020), we examined this relationship in our MG sample; however, no correlation was found implicating that age at sampling does not affect the association (Figure 3C).

### 3.6 CD4 T Cells and Clinical Characteristics

Finally, we performed Cox regression analyses to correct for known confounders. Univariable analysis identified the factors CD4 T frequency and disease duration > 12 months (Table 3) as associated with the outcome. We thereafter constructed a multivariable Cox regression model including covariates with *p* < 0.2 in the univariable analyses. While both age at sampling and disease duration > 12 months had nominally significant *p*‐values, the only statistically significant predictor of time to minimal disease manifestation, after correction for multiple testing, was elevated CD4 T cell frequency (*p* = 0.0014, HR = 0.94, 95% CI: 0.91–0.97). This hazard ratio of 0.94 indicates that for every 1% increase in CD4 T cell frequency, the likelihood of achieving minimal disease manifestation decreases by 6%.

**TABLE 3:** Cox regression analysis of predictors for minimal disease manifestation.

	Univariable			Multivariable			Adj *p*
	HR	95% CI	*p*	HR	95% CI	*p*	
**Age at sampling**	**1** **.02**	**1.00–1.05**	**0.07**	**1.04**	**1.00–1.08**	**0.047**	
Male sex	0.94	0.44–2.0	0.87				
**Late onset MG**	**1.74**	**0.80–3.81**	**0.17**	1.18	0.41–3.42	0.76	
Ocular MG	1.15	0.27–4.87	0.85				
QMG score	0.98	0.92–1.06	0.67				
Thymectomy	0.63	0.28–1.41	0.26				
**Disease duration > 12 months**	**0.60**	**0.29–1.25**	**0.17**	**0.36**	**0.15–0.84**	**0.02**	
Any current IST	1.04	0.50–2.14	0.92				
Current steroids	1.24	0.55–2.77	0.59				
Current IVIG	1.06	0.20–2.80	0.91				
Current azathioprine	0.98	0.35–2.74	0.97				
Current rituximab	1.29	0.18–9.5	0.80				
CD4 T cells/nL	0.86	0.35–2.12	0.74				
**CD4 T cells (% of T cells)**	**0.96**	**0.93–0.99**	**0.004**	**0.94**	**0.91–0.97**	**0.0001**	**0.0014**

*Note:* Months to minimal disease manifestation in myasthenia gravis patients (*n* = 52) with active disease at baseline was modeled using Cox regression. Predictors of particular interest are in blod: those with *p*‐value < 0.2 in univariable analyses, which were included in the multivariable model, and predictors that had a *p*‐value < 0.05 in the multivariable model. A hazard ratio (HR) < 1 indicates that the predictor reduces the likelihood of having minimal disease manifestation within one year. *p*‐values were adjusted for 14 tests using the Bonferroni method and only those with adjusted *p*‐values (Adj *p*) < 0.05 are presented.

Abbreviations: CI, confidence interval; IST, immunosuppressive treatment; IVIG, intravenous immunoglobulin; QMG, quantitative myasthenia gravis.

## 4 Discussion

In this exploratory study investigating the correlation between peripheral blood lymphocyte phenotypes and clinical characteristics of MG, we identified changes that suggest a potential role for CD4 T cell frequency in disease management. Notably, untreated MG patients exhibited differences in both the B and T cell compartments compared to age appropriate reference populations, with a particularly striking increase in CD4 T cell frequency, which was remarkably stable over time. More importantly, CD4 T cell frequency emerged as a potential prognostic marker, correlating with the likelihood of achieving minimal disease manifestation within one year of sampling.

Alterations in CD4 T cell frequencies have been linked to several inflammatory diseases. A reduced CD4/CD8 ratio has repeatedly been associated with immunosenescence, aging, and increased morbidity (Wikby et al. 2008; Serrano‐Villar et al. 2014). In contrast, an elevated CD4/CD8 T cell ratio in bronchoalveolar lavage and cerebrospinal fluid is a supportive diagnostic marker of sarcoidosis, and an increased proportion of CD4 T cells are found in the granulomas (Chopra et al. 2016; Nordström et al. 2020). Increased CD4 T cell frequencies and CD4/CD8 ratio have previously been reported in MG patients with generalized disease. Further, increased CD4 T cell frequencies were observed in MG patients with a recent exacerbation compared to patients with minimal disease manifestation (Hu et al. 2020; Bi et al. 2023). Moreover, studies using the murine experimental autoimmune myasthenia gravis (EAMG) model suggest that CD4 and not CD8 T cells are instrumental in the pathogenesis of MG (Wang et al. 1999). While most immune modulatory treatments resulted in a reduction in lymphocyte population concentrations in our data, most markedly CD4 T cells, CD4 T cell frequencies remained stable over time, consistent with a genetically determined trait. Genetic control of CD4 frequency has been described and linked to autoimmune disease (Hall et al. 2000; Ahmadi et al. 2001; Ferreira et al. 2010).

In the present study, we observed that an increased CD4 T cell frequency in peripheral blood of MG patients was associated with a lower likelihood of reaching minimal disease manifestation within one year. Mechanistically, this may reflect an extensive antigen‐driven expansion of autoreactive CD4 T cells that support a robust and long‐lasting humoral autoimmune response and are perhaps less amenable to intrinsic feedback mechanisms in the immune system and therapeutic immune modulation. Indeed, our data suggest a selective expansion of central memory Th1 cells in MG compared to healthy individuals, suggestive of chronic antigen‐driven expansion of autoreactive T cells. A high CD4 percentage may alternatively be caused by upstream inflammatory triggers that also influence MG disease activity. However, assuming that CD4 T cells are drivers of disease, it is likely a subset of this large and heterogeneous lymphocyte population that is important. Whilst some studies are performed to define key effector mechanisms in MG, the main focus of this study is the utility of well‐established clinical laboratory analyses (Hall et al. 2000; Ahmadi et al. 2001; Ferreira et al. 2010). Although ROC‐curve analysis indicated only moderate accuracy, this information may still influence clinical decisions on follow‐up and treatment intensity, and CD4 T cell frequencies may be used as one of several biomarkers in future prognostic algorithms.

Limitations of the study include the retrospective design utilizing secondary clinical data, whereby results can only be regarded as hypothesis‐generating. Still, by linking the two databases, we were able to capture virtually all patients analyzed with flow cytometry at the Karolinska University Hospital Laboratory, which serves several hospitals in the Stockholm County, thus minimizing a source of potential bias. It is important to note, however, that immunophenotyping was done in two categories of patients: those with new onset disease and those with refractory symptoms despite ongoing immune modulation to be switched to rituximab. An additional limitation is that the reference population was not recruited to be controls in this study; the data was only available to us in the form of summary statistics.

The utility of CD4 T cell frequencies as a prognostic biomarker needs to be validated in a prospective study before application in clinical practice. However, before designing the protocol for such a study, the observed association with MG prognosis should be confirmed and further characterized in an independent dataset. The quantification of CD4 T cells is a widely available analysis that is subjected to rigorous quality assessments in clinical practice, which will facilitate efforts to gather laboratory data from several centers.

In sum, we show that an analysis that is well established in clinical routine for determining CD4 T cells may serve a role as a prognostic marker in MG, but further studies to corroborate the results are needed before more firm conclusions can be made.

## Author Contributions

**Hannes Lindahl:** conceptualization, writing – original draft, methodology, visualization, writing – review and editing, formal analysis, project administration, data curation. **Malin Petersson:** data curation, writing – review and editing, methodology. **Sara Lind Enoksson:** data curation, writing – review and editing. **Fredrik Piehl:** funding acquisition, writing – review and editing, supervision. **Susanna Brauner:** supervision, project administration, writing – review and editing, funding acquisition, conceptualization, data curation.

## Ethics Statement

The study was approved by the Regional Ethics Review board of Stockholm, Sweden (Dnr 2016/828–31). The study was conducted according to the RECORD guidelines.

## Conflicts of Interest

Susanna Brauner has, not related to this study, received non‐restrictive research grants from UCB Pharma and Janssen. Fredrik Piehl has received research grants from Janssen, Merck KGaA and UCB, and fees for serving on DMC in clinical trials with Chugai, Lundbeck and Roche, and preparation of expert witness statement for Novartis.

## Peer Review

The peer review history for this article is available at https://www.webofscience.com/api/gateway/wos/peer‐review/10.1111/jnc.70126.

## Supporting information


Data S1.

## Data Availability

Raw data is provided in a Supporting Information data file. Due to ethical considerations, certain patient characteristics are omitted from this file to avoid identification of individuals.
