# Supplementary material for: Extensive Clinical Flow Cytometric Lymphocyte Phenotyping in Myasthenia Gravis: A Single‐Center Study
Source: J Neurochem. 2025 Jun 17;169(6):e70126. doi: 10.1111/jnc.70126 (PMC12172393; doi:10.1111/jnc.70126)
Supplement: Supplementary file 1 — Data S1. [file JNC-169-0-s001.pdf]

Extensive clinical flow cytometric lymphocyte phenotyping in Myasthenia Gravis: A single-center study.
Authors: Hannes Lindahl, Malin Petersson, Sara Lind Enoksson, Fredrik Piehl, and Susanna Brauner

SUPPLEMENTARY MATERIAL

Table S1 Flow cytometry results of untreated MG including subtypes

Table S2 Characteristics of patients in Figure 3

Figure S1 Gating strategy

Figure S2 Flow cytometry results in relation to current MG treatment

Extensive clinical flow cytometric lymphocyte phenotyping in Myasthenia Gravis: A single-center study.
Authors: Hannes Lindahl, Malin Petersson, Sara Lind Enoksson, Fredrik Piehl, and Susanna Brauner

Table S1 Flow cytometry results of untreated MG including subtypes

	All			EOMG			LOMG		
	Median	IQR	Mean	Median	IQR	Mean	Median	IQR	Mean
T cells/uL	1205	903 – 1530	1234	1220	925 - 1790	1338	1205	903- 1530	1220
CD4 T cells/uL	830	653 - 1108	883	905	615 - 965	836	795	665 – 1138	896
CD8 T cells/uL	280	188 - 413	374	365	245 - 863	504	255	180 – 358	338
DP T cells/uL	21.3	10.5 - 32.3	24.7	21.5	19.6 - 43.7	31.9	18.3	10.6 - 33.6	25.6
B cells/uL	140	83 - 250	192	160	115 – 245	243	120	70 - 245	179
NK cells/uL	225	140 - 280	224	230	115 – 330	232	230	145 - 280	227
CD4, % of T cells	75	65.8 - 80.3	72.2	66	60.3 71.3	64.3	78	71 - 82.8	74.5
Naïve, % of CD4	43	24.5 - 49.5	39	47.5	43.5 - 53.5	45.9	38	22 – 49	36.9
EM	14	11.5 - 18	14.7	14.5	12 - 20.3	15.5	14	10 – 18	14.5
CM	41	34 - 54	44.1	34.5	31.8 - 41	36	46	35 – 58	46.4
EMRA	1	0.5 - 2.5	2.5	1	0.5 - 5.3	3.1	1	0.5 – 2	2.4
Activated	2	1 – 3	2.3	1	0.5 - 2.8	1.4	2	1 – 3	2.5
Th1 EM	5.8	3.9 - 7.8	6.1	6.73	5.1 - 7.7	6.2	5.4	3.5 - 8.5	6
Th1 CM	11.9	8.7 - 14.6	12	11	10 - 13.1	10.8	12.4	8.3 - 16.2	12.4
Th2 EM	1.6	1.16 - 2.4	2.1	2.2	1.1 - 2.9	2.1	2.1	1.5 - 2.9	2.1
Th2 CM	10.8	7.3 - 14.7	11.4	7.8	5.5 - 11.8	8.2	11.8	7.9 - 15.6	11.5
Th17 EM	2.1	1.4 - 2.8	2.1	1.8	1.6 – 3	2.1	1.4	0.96 - 2.8	2.1
Th1 CM	11	7.7 - 13	10.8	6.6	5.4 - 10.5	8	11.2	8.8 - 15.8	12.4
CD8, % of T cells	20.5	15.8 - 29.3	24	27.5	23.5 - 35.3	30.4	19.4	12.8 - 25.3	22.2
Naïve, % of CD8	23	13 - 37.5	25.7	37	28.8 - 42.8	34.6	21	8 – 31	23
EM	23	18 - 27.5	25.1	24	23 - 41.5	27.9	23	16 - 27	24.3
CM	12	8.5 - 19	16	11.5	6.3 - 15.3	11.8	12	9 – 24	17.3
EMRA	30	18 - 46.5	33.3	26	15 - 33.8	25.8	32	18 - 47	35.6
Activated	5	1.5 - 10	6.7	3	0.63 - 5.8	4.1	6	2 – 10	7.4
DP T, % of T cells	1.9	1.1 - 3.1	2.1	2.1	1.5 - 2.5	2.4	1.8	0.98 - 3.3	2.2
Transitional, % of B cells	1	0.5 - 2	2.1	1.5	0.63 - 11	4.4	1	0.5 – 2	1.4
Naïve	81.5	62.8 - 84.5	69.7	72.5	64.5 - 80.5	72.5	83	58 – 86	68.4
Preswitched memory	4	2 - 7.1	7.7	5	2.3 - 14	7.1	4	2 – 9	7.9
Switched memory	9.5	6.5 - 19.3	14.2	12	3.6 - 17	10.9	9	7 – 28	15.5
Plasmablasts	0.5	0.5 - 1	0.6	0.6	0.5 - 0.93	0.7	0.5	0.5 – 1	0.84

Peripheral blood flow cytometry results from untreated myasthenia gravis (MG) patients (n = 42 for T, B, and NK cell concentration; n = 35 for CD4 and CD8 T cells and their memory and polarization states; and n = 24 for B cell subsets). The 42 MG patients include 1 patient with thymoma associated MG (TAMG), 9 with early onset MG (EOMG), and 32 with late onset MG (LOMG), of which the latter two groups are presented separately. Double positive (DP) T cells are $CD4^{+}$ $CD8^{+}$. T helper (Th) 1 is defined as CD4 T cells that are $CXCR3^{+}$ $CCR6^{-}$, Th2 as $CXCR3^{-}$ $CCR6^{-}$, and Th17 as $CXCR3^{-}$ $CCR6^{+}$. Activated T cells are defined as HLA-DR^{+} $CD38^{+}$. Preswitched memory B cells are defined as $CD19^{+}$ cells that are IgD^{+} $CD27^{+}$, plasmablasts as $CD19^{+}$ cells that are $IgM^{-/+}CD38^{++}$, transitional B cells as $CD19^{+}$ cells that are IgM^{++} $CD38^{++}$. n, number of participants; IQR, interquartile rangel; EM, effector memory T cells; CM, central memory T cells; EMRA, $CD45RA^{+}$ EM T cells

Table S2 Characteristics of patients in Figure 3

	**CD4 low (n = 31)**		**CD4 high (n = 21)**	
Female, n (%)	17 (55)		7 (33)	
Age at sampling, median (IQR)	62 (47 - 73)		67 (57.5 - 73)	
Subtype, n (%)				
EOMG	14 (45.2)		1 (4.8)	
LOMG	13 (41.9)		18 (85.7)	
TAMG	4 (12.9)		2 (9.5)	
QMG score, median (IQR)				
At sampling	7 (5 - 10)		8.5 (7 - 13)	
After 12 m	2 (0 - 7)		5 (1 - 6.5)	
Disease duration in y, median (IQR)	1.4 (0.3 - 14.5)		0.8 (0.1 - 9.0)	
Treatment and duration range in days	n (%)	Range	n (%)	Range
None	10 (32.3)		13 (61.9)	
Glucocorticoids	9 (29)	7d – 3y	1 (4.8)	NA
IVIG	3 (9.7)	6d – 1y	2 (9.5)	6d – 10d
Azathioprine	3 (9.7)	6y – 12y	2 (9.5)	NA – 11y
Mycophenolate mofetil	0		1 (4.8)	5y
Multiple	6 (19.4)	7d – 1y	2 (9.5)	6m – 2y

IQR, interquartile range; EOMG, early onset MG; LOMG, late onset MG; TAMG, thymoma associated MG; QMG, quantitative MG; IVIG, intravenous immunoglobulin; d, days; m, months; y, years; NA, not available; n, number of participants

Extensive clinical flow cytometric lymphocyte phenotyping in Myasthenia Gravis: A single-center study.
Authors: Hannes Lindahl, Malin Petersson, Sara Lind Enoksson, Fredrik Piehl, and Susanna Brauner

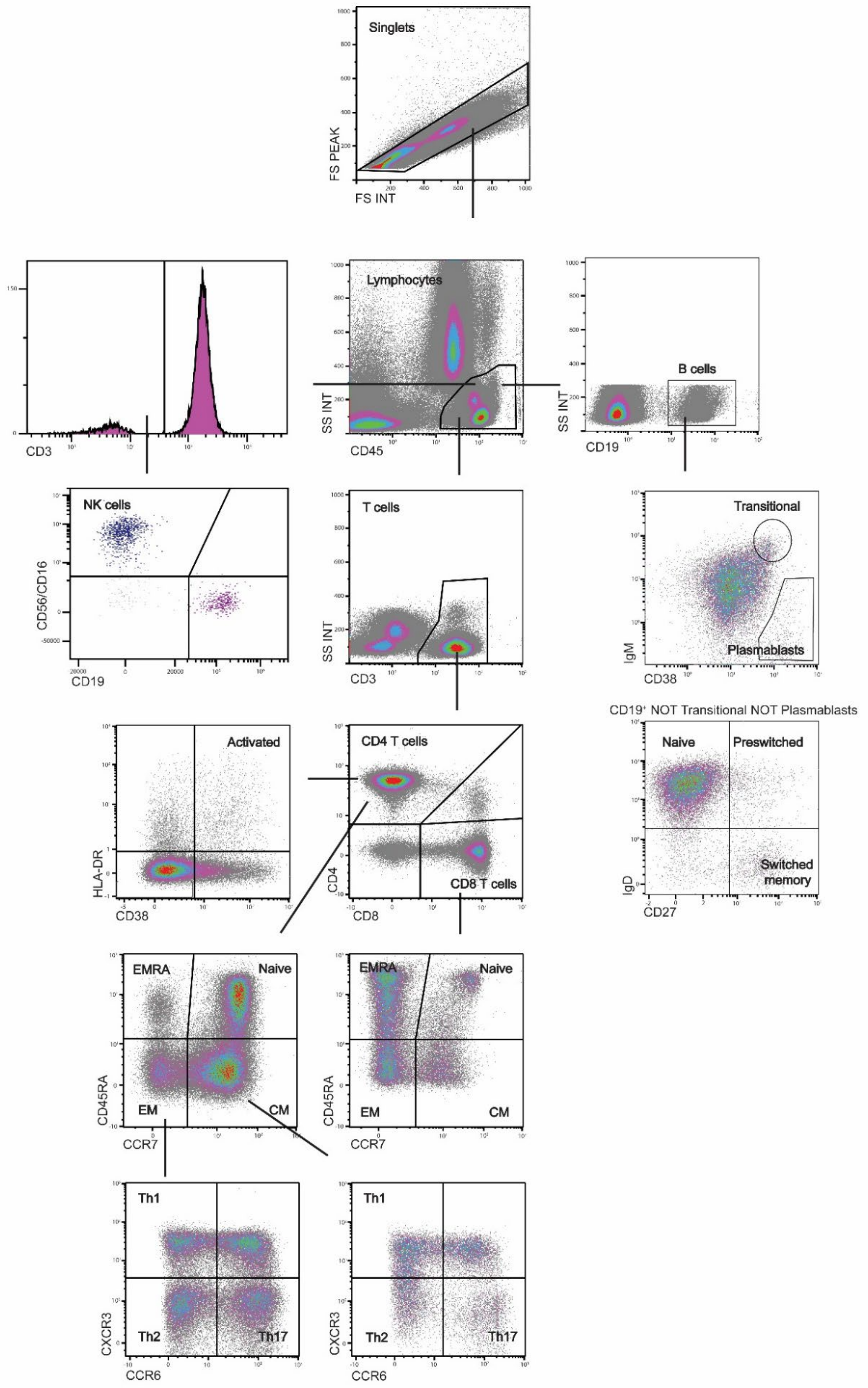

Figure S1. **Gating strategy**

Extensive clinical flow cytometric lymphocyte phenotyping in Myasthenia Gravis: A single-center study.
Authors: Hannes Lindahl, Malin Petersson, Sara Lind Enoksson, Fredrik Piehl, and Susanna Brauner

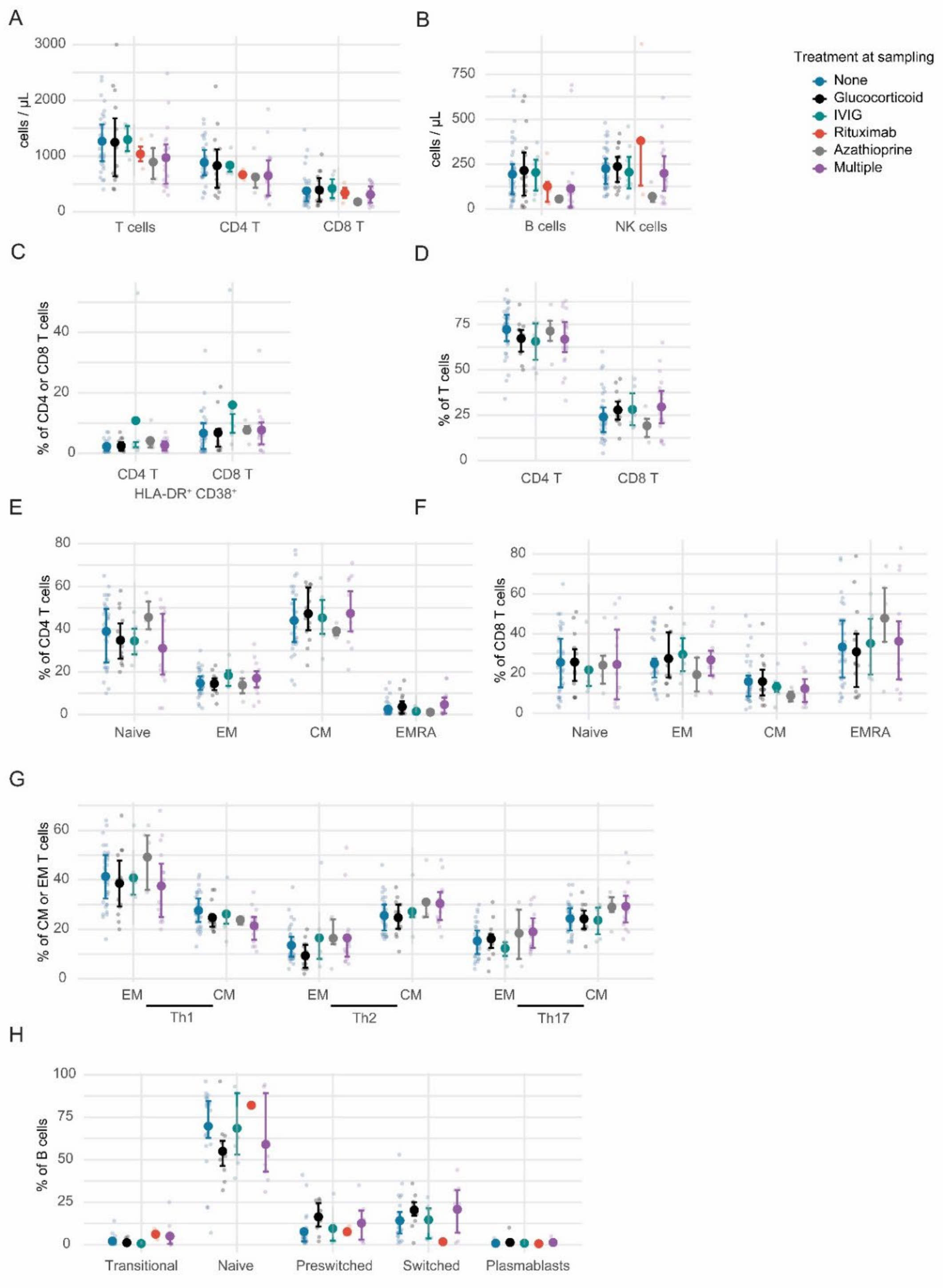

Figure S2. **Flow cytometry results in relation to current MG treatment.** Data from blood samples taken during no immune modulatory treatment, treatment with a single drug or combination treatment (multiple) are plotted to illustrate the effects of specific treatments on lymphocyte subsets. Median and interquartile range are indicated. Untreated were compared to the different treatment categories using Mann-Whitney U-test, however after adjustment using the Bonferroni method nothing remained statistically significant. IVIG, intravenous immunoglobulin; EM, effector memory T cells; CM, central memory T cells; EMRA, CD45RA^{+} Effector memory T cells
